# Development of an Extracorporeal Perfusion Device for Small Animal Free Flaps

**DOI:** 10.1371/journal.pone.0147755

**Published:** 2016-01-25

**Authors:** Andreas M. Fichter, Lucas M. Ritschl, Anna Borgmann, Martin Humbs, Peter B. Luppa, Klaus-Dietrich Wolff, Thomas Mücke

**Affiliations:** 1 Department of Oral and Maxillo-Facial Surgery, Technische Universität München, Klinikum Rechts der Isar, München, Germany; 2 Institute for Clinical Chemistry and Pathobiochemistry, Technische Universität München, Klinikum Rechts der Isar, München, Germany; University Hospital Oldenburg, GERMANY

## Abstract

**Background:**

Extracorporeal perfusion (ECP) might prolong the vital storage capabilities of composite free flaps, potentially opening a wide range of clinical applications. Aim of the study was the development a validated low-cost extracorporeal perfusion model for further research in small animal free flaps.

**Methods:**

After establishing optimal perfusion settings, a specially designed extracorporeal perfusion system was evaluated during 8-hour perfusion of rat epigastric flaps followed by microvascular free flap transfer. Controls comprised sham-operation, ischemia and *in vivo* perfusion. Flaps and perfusate (diluted blood) were closely monitored by blood gas analysis, combined laser Doppler flowmetry and remission spectroscopy and Indocyanine-Green angiography. Evaluations were complemented by assessment of necrotic area and light microscopy at day 7.

**Results:**

ECP was established and maintained for 8 hours with constant potassium and pH levels. Subsequent flap transfer was successful. Notably, the rate of necrosis of extracorporeally perfused flaps (27%) was even lower than after *in vivo* perfusion (49%), although not statistically significant (*P* = 0,083). After sham-operation, only 6% of the total flap area became necrotic, while 8-hour ischemia led to total flap loss (98%). Angiographic and histological findings confirmed these observations.

**Conclusions:**

Vital storage capabilities of microvascular flaps can be prolonged by temporary ECP. Our study provides important insights on the pathophysiological processes during extracorporeal tissue perfusion and provides a validated small animal perfusion model for further studies.

## Introduction

Prolonged vital storage of organs by temporary extracorporeal perfusion (ECP) was first demonstrated by Alexis Carrel [1] in the 1930s and is today routinely used for the vital storage of isolated organs [2–4]. As opposed to isolated organs, however, composite flaps (or extremities) are composed of different tissues with a varying tolerance towards ischemic damage. While bone, tendons skin and fat usually tolerate intervals of prolonged ischemia quite well, muscle, blood vessels and neuronal tissue have a low tolerance towards ischemia [5]. The warm ischemia time, defined as the time a certain tissue can tolerate the lack of oxygen without special measures like cooling, should not exceed six hours in composite tissues [6]. A second reason why flap perfusion differs from organ perfusion is the fact that microvascular free flaps are no isolated, confined organs. Instead, flaps are harvested from their environment resulting in a large wound surface, promoting microbial contamination and excessive bleeding during prolonged extracorporeal perfusion. Nevertheless, existing studies indicate that ECP may hold the potential for prolonged vital storage of composite tissues [7–10], but fundamental questions, like optimal perfusion pressure and flow settings, oxygen/ carbondioxide ratio, perfusate temperature and the composition of an ideal perfusate still remain unanswered. Moreover, clinical use of such a system has not yet been established for free flap perfusion.

Studies aimed at the perfusion of living tissues depend on expensive and resource intensive large animal models since perfusion is usually achieved using commercially available systems borrowed from human cardiac bypass circuits. Due to the small size and low blood volumes, these circuits are not suited for small animal free flaps [11]. Our study group could already demonstrate the suitability of an extracorporeal perfusion device in the training of microsurgeons to simulate realistic conditions in cadavers [12]. The objective of the present study was to develop and evaluate a low-cost extracorporeal perfusion device for the prolonged vital storage of small animal free flaps that should serve as a model for future free flap perfusion studies.

## Materials and Methods

All animals were treated and housed in accordance with the EU-guidelines. The study was approved by the regional government (Regierung von Oberbayern, AZ55.2-1-54-253-86-08) and was conducted in accordance with the German Animal Welfare Act. A total of 64 male Fischer-344 rats (250–300g, Fa. Charles River, Kißlegg, Germany) were used. Food and water were provided *ad libitum*. All surgical procedures were performed under intravenous general anesthesia [ketamine 100mg/kg (Narketan^®^, Fa. Vétoquinol GmbH, Ravensburg, Germany) und xylazine 5 mg/kg (Rompun^®^, Fa. Bayer Vital GmbH, Leverkusen, Germany)] and aseptic conditions as described elsewhere [13], and all efforts were made to minimize suffering.

### Preliminary Studies

In a first step, physiological blood flow values were established at 0.84 ± 0.6 ml/min (femoral artery) and 1.29 ± 0.63 ml/min (femoral vein), respectively, using transit time flowmetry in 12 rats. With regard to long-term perfusion, the new system was tested for hemolysis using a closed circuit with no organ connected at a pace of 5 ml/min, filled with heparinized rodent whole blood (1 IE heparin/ml) drawn from 8 rats. While remaining within the reference range of the animal model [14], lactate and lactate dehydrogenase activity showed a statistically significant increase within 4 hours (*P* = 0.039), consistent with progressive hemolysis.

### The Perfusion System

**Fig 1:**
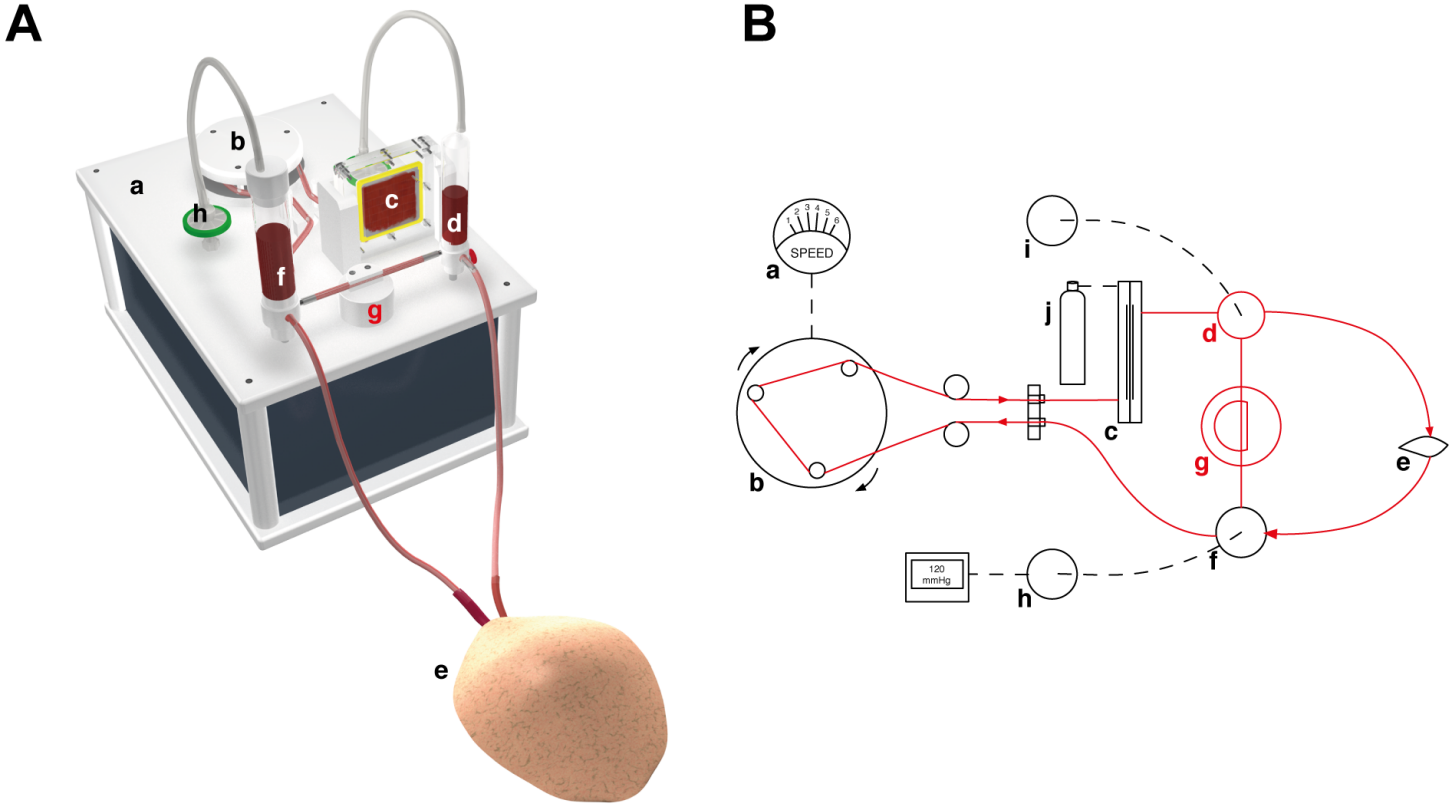
The perfusion system. (A) 3D rendering of the perfusion system used in this study. (B) Schematic drawing of the experimental setup. A motor (a) driven roller pump (b) pumps heparinized autologous blood through an oxygenator membrane (c), connected to an oxygen cylinder (j). The oxygenized blood is flows into the flap (e), while the venous return is collected in a reservoir (f) connected to a pressure gauge (h). If the pressure in the system surpasses a certain treshold, the pressure equalizes by opening a shunt (g) between arterial (d) and venous reservoirs. (i) Bubble trap.

The setup of the perfusion system used for the *in vivo* studies is presented in Fig 1. The system was configured in a way that all individual parts (tubes, connectors, reservoirs, membrane oxygenator) that came in contact with blood could be sterilized and assembled and filled with blood under sterile conditions. The perfusion system consisted of a roller pump (Ing. Büro Humbs, Valley, Germany), a membrane oxygenator (type Oxyphan PP50/280, Fa. Membrana GmbH, Wuppertal, Germany) that worked with both ambient air and pure oxygen, an arterial and a venous reservoir. The venous reservoir was connected to a vacuum and could generate an adjustable negative pressure to the venous system. Both reservoirs served as a bubble trap. The blood pressure before the flap was continuously measured (Panasonic Electric Works SUNX Co., Ltd. Kasugai City, Aichi, Japan). A pressure controlled shunt between arterial and venous blood reservoir was implemented to avoid uncontrolled pressure spikes. The tubing system consisted of platinum-curing silicones (USP class IV, FDA-approved) and had an inner diameter of 0.8 mm and a total length of 2.5 meters. All materials were steam sterilized before each usage.

Thirty minutes before initiation of the extracorporeal tissue perfusion, the tubing system was rinsed with heparinazed Ringer’s solution (10ml Ringer, 100 IE Heparin) at 5 ml/min. The rinsing solution was then replaced by the perfusate. Subsequently, the femoral vessels of the raised flap were connected to the perfusion unit using modified IV catheters (Vasofix^®^ Braunüle^®^ 20 G and 18 G, Fa. B. Braun Melsungen AG, Melsungen, Germany). Extracorporeal perfusion was commenced at 2 ml/min (corresponding to high-normal physiological values) and was maintained at the same speed for 8 hours. An upper pressure limit was programmed at 130 mmHg, also corresponding to high physiological values [15].

To minimize a detrimental effect of progressive hemolysis, the perfusate was replaced after 4 hours and if (1) the filling volume reached a critical value of ≤ 1 ml in the venous reservoir, or if (2) the closely monitored pH fell below 7.300.

### Perfusate Composition and Temperature

To improve rheological properties and compensate for short supply, diluted (40% Ringer’s solution and 10% hydroxyethyl starch), heparinized (10 IE Heparin/ml blood) rodent whole blood was used as perfusate. A plasma expander (hydroxyethyl starch, HES, Volulyte 6%, Fa. Fresenius Kabi Deutschland GmbH, Bad Homburg v.d.H., Germany) was added to prevent excessive edema formation. The room temperature in the laboratory was controlled at 21 ± 1°C by an air-conditioning system. The perfusate temperature was allowed to equilibrate to room temperature (21 ± 1°C) prior to perfusion, where it remained constant throughout the extracorporeal perfusion period without additional control mechanisms. The composition of the perfusate was kept unchanged throughout the experiment and was closely monitored during extracorporeal tissue perfusion. Therefore, blood samples were drawn immediately after preparation of the perfusate (base value), as well as every 15–30 minutes during the 8-hour tissue perfusion. Blood gas and laboratory-chemical analysis (RAPIDLAB^®^ 348, Siemens Sector Healthcare, Erlangen) were performed with detection of pH-value, potassium (K^+^), oxygen saturation (SO_2_), oxygen partial pressure (pO_2_), carbon dioxide partial pressure (pCO_2_), sodium bicarbonate buffer (HCO_3_^–^) and hemoglobin level (Hb).

### Surgical Groups

In 32 rats, the epigastric flap was raised based on the superficial epigastric vessels in the dimensions of 4 x 7 cm, as described in detail by Strauch and Murray [16]. The experimental groups are listed in Table 1. Another 32 rats served as either recipient animals for microvascular free flaps or as blood donors.

**Table 1:** Experimental protocol.

	Surgical group	Perfusion (hours)	Ischemia (hours)	Flap transfer	Sample size
C1	Control group 1: „*sham*-operation“	0	0	–	8
C2	Control group 2: ischemia	0	8	+	8
C3	Control group 3: *in vivo* perfusion	8	0	+	8
ECP	Extracorporeal perfusion	8	0	+	8

**Fig 2:**
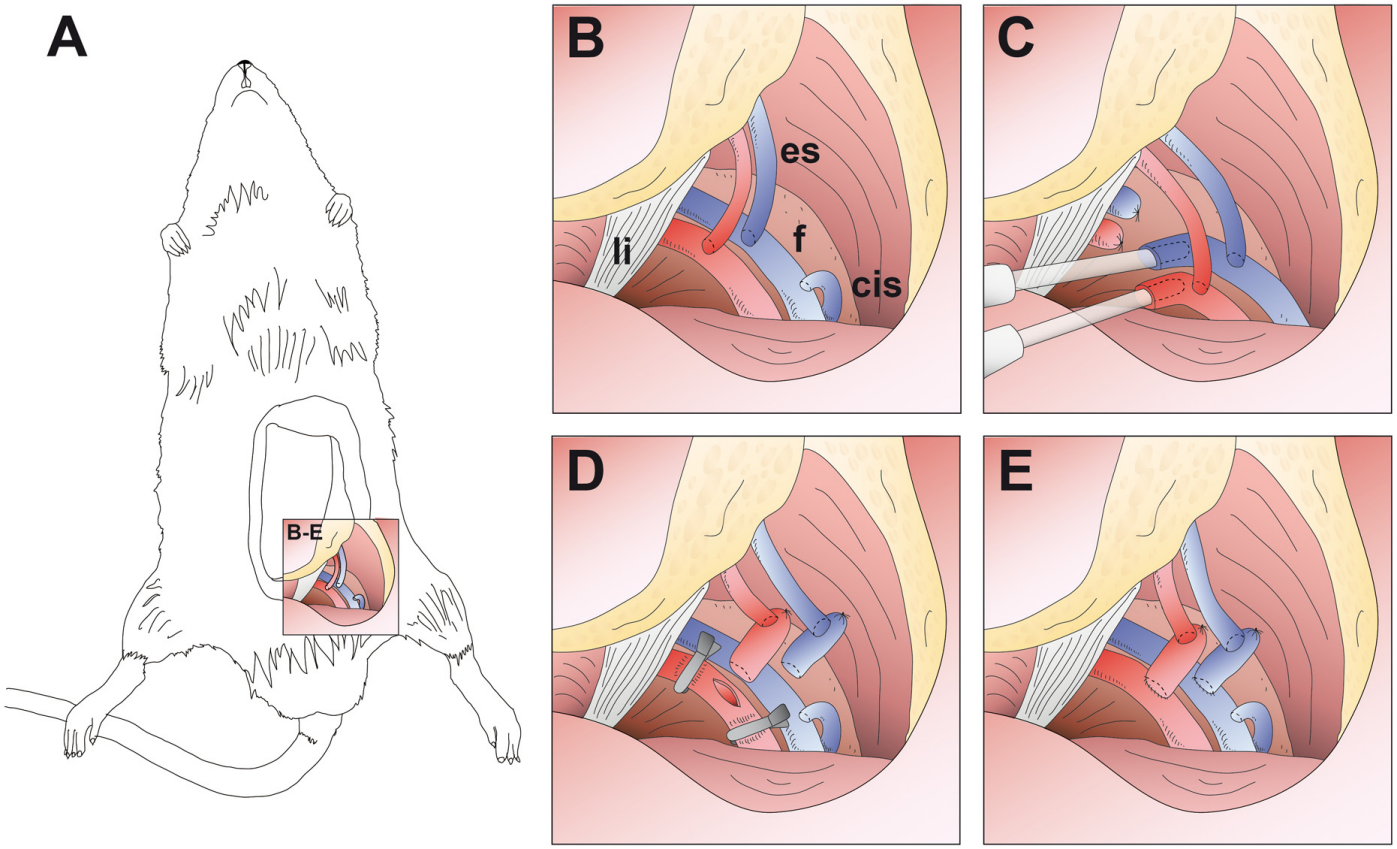
Experimental setup (schematic drawing). (A) Overview image of a rat with raised epigastric flap perfused solely by the superficial epigastric vessels. (B-E) Detail images. (B) Operative situs with superficial epigastric vessels (es), femoral vessels (f), inguinal ligament (li) and superficial circumflex iliac vein (cis). (C) Canullated femoral vessels connected to the extracorporeal perfusion device. (D) After 8-hour continuous extracorporeal perfusion, femoral vessels are ligated distally to the epigastric vessels and severed proximally to the epigastric vessels. Subsequently, the flap is transferred to a recipient rat and the femoral vessel stumps are anastomosed end-to-side to the femoral vessels of the recipient animal. (E) Situs after successful microvascular flap transfer.

In group C1 (sham-operation), flaps were sutured back to the wound bed immediately after flap raise and animals were allowed to wake. In group C2 (ischemia), flaps were stored at room temperature for 8 hours. Subsequently, a second (recipient) rat was anesthetized and the epigastric flap was raised as described above and discarded. The ischemic flap was then transferred to the recipient animal, vessels were anastomosed end-to-side to the femoral vessels, the skin island was sutured into the wound bed and the recipient animals were allowed to wake. Donor animals were euthanised by intravenous lethal injection [2 ml per animal] of a combination of 200 mg Embutramid, 50 mg Mebezonium and 5 mg Tetracain per ml (T61^®^, Intervet, Unterschleißheim, Germany). In group C3, *in vivo* perfusion was maintained for 8 hours. Subsequently, the *in vivo* perfused flaps were transferred to a recipient rat. The experimental setup of group ECP (extracorporeal perfusion) is depicted in Fig 2. In this group, the nutritive vessels of the flap were connected to the perfusion device and extracorporeal perfusion was maintained for 8 hours. Subsequently, flaps were disconnected and transferred to a recipient rat. For postoperative analgesia, the rats received buprenorphine (50 μg/kg s.c.; Temgesic^®^; Essex Pharma, Germany) directly after waking up, as well as every 12 hours for 3 days.

### Assessment of Flap Perfusion

In addition to observing flap color and capillary refill, measurement of tissue oxygen saturation (SO_2_ in %), hemoglobin level (Hb, in AU, arbitrary units), blood flow (flow, in AU) and velocity (velocity, in AU) was noninvasively performed using comined laser Doppler flowmetry and remission spectroscopy (O2C, equipped with an LF-2 probe, Lea Medizintechnik, Giessen, Germany). This technique has been described in detail elsewhere and is an established procedure for the assessment of free flaps viability [17]. In all surgical groups, O2C was performed preoperatively (base value) and at day 7. In group C2, C3 and ECP, additional O2C measurements were conducted after flap raise and after wound closure. During *in vivo* (C3) and extracorporeal perfusion (ECP), O2C was performed every 30 minutes. All measurements were performed in the center of the flap with equal surface pressure and full contact between probe and skin.

Indocyanine-Green (ICG) fluorescence angiography was performed in groups C3 and ECP during *in vivo* and extracorporeal perfusion, respectively. The fluorescence signal was recorded using a mobile near infrared (NIR) fluorescence camera (PDE Photodynamic Eye, Pulsion Medical Systems SE, Feldkirchen, Germany). Flow analysis was performed with IC-CALC (Version 2.0, Pulsion Medical Systems SE, Feldkirchen, Germany).

### Planimetric Measurement of Necrotic Areas

Flap healing was documented using a digital SLR camera (type Nikon D700, Fa. Nikon Corp., Chiyoda, Tokyo, Japan) mounted in a perpendicular direction to the flap with a tripod at day 7. Pictures were analyzed with respect to vital and necrotic areas. Therefore, total flap area and necrotic areas were manually circumscribed with the help of a graphic tablet and pen and the cross-sectional area was calculated using ImageJ [18].

### Structural Analysis

After documentation of the necrotic area, rats were euthanized as described above, while still in deep anesthesia. Epigastric flaps were harvested and fixed in a 4% formalin solution. The flap samples were then embedded in paraffin, sectioned at 5 μm and stained with hematoxylin and eosin for histological evaluation under light microscopy.

### Statistics

The SPSS software package (SPSS 22, SPSS Inc., Chicago, IL, USA) was used for statistical analysis. For rate of necrosis, the Kuskal-Wallis test was chosen to determine significant differences (*P* < 0.05) between groups. If a significant difference was detected, the Mann-Whitney U test was performed to compare the groups in pairs. Wilcoxon’s test was used to compare parameters inside a study group. The multiple test problem was not taken into account. All data are presented as mean ± standard deviation. Differences were considered statistically significant for a two-sided exact *P* value of less than 0.05. All observations were independently evaluated by two investigators blinded to the experimental groups.

## Results

### Assessment of the Perfusate Composition

Laboratory-chemical values measured after routine replacement of the perfusate after 4 hours (perfusion hours 4–8) closely resembled those measured in the first 4 hours of perfusion. Since in the second half of the perfusion period, laboratory-chemical assessment, however, was only sporadically performed these data were not considered for statistical analysis (Table 2). In 2 cases, the perfusate had to be changed prematurely at around 3.5 hours since the filling volume had sunk under the critical value and since the pH had fallen below 7.300, respectively.

**Table 2:** Statistical analysis of laboratory-chemical changes in the perfusate.

	Parameter							
	pH	SO_2_	pO_2_	pCO_2_	HCO_3_^–^	BE	K^+^	Hb
Median	7.464	98.7	118.9	17.6	13.4	–10.3	4.34	4.2
Median 4h	7.363	99.3	131.7	7.7	5.1	–18.2	5.61	4.5
Minimum	7.338	93.4	63.0	5.9	2.4	–18.4	3.33	2.0
Maximum	7.515	99.8	262.2	26.4	18.6	–4.5	6.45	10.3
Difference 0–4h	0.101	–0.6	–12.8	9.9	8.3	7.9	–1.27	–0.3
*P* values 0–4h	0.063	0.031*	0.031*	0.031*	0.031*	0.031*	0.031*	0.563

Comparison between preoperative base values (0) and values obained from the venous reservoir after 4 hours of extracorporeal tissue perfusion. Wilcoxon’s test was used for statistical analysis. Statistically significant differences (*P* < 0.05) between time points are marked with an asterisk (*).

**Fig 3:**
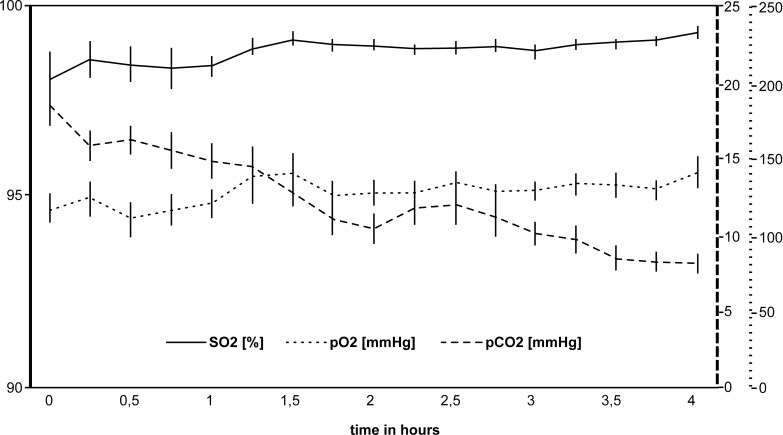
Changes in the blood gases during extracorporeal perfusion. The use of a membrane oxygenator yielded high oxygen levels (SO_2_) and oxygen partial pressures (pO_2_). Carbon dioxide partial pressure (pCO_2_) decreased continuously during the first 4 hours of extracorporeal perfusion. The error bars indicate the standard error.

Changes in the blood gas parameters are illustrated in Fig 3. Oxygen saturation and oxygen partial pressures showed high-normal values throughout the extracorporeal perfusion (base values SO_2_ 98.7%, pO_2_ 118.9 mmHg). Both SO_2_ (*P* = 0.016) and pO_2_ (*P* = 0.008) increased significantly during the first 1.5 hours and stayed constantly high for the remaining time (SO_2_ 99.3 mmHg, pO_2_ 131.7 mmHg after 4 hours). Carbon dioxide partial pressures (17.6 mmHg, median) started below reference values (32.6–41.0 mmHg) and further decreased during the following 4 hours (7.7 mmHg, *P* = 0.031).

Oxygen partial pressures in the arterial reservoir (128.4 mmHg) were significantly higher than those measured in the venous reservoir (34.2 mmHg). The difference between arterial (98.9%) and venous (65.2%) oxygen saturation was 33.7%.

**Fig 4:**
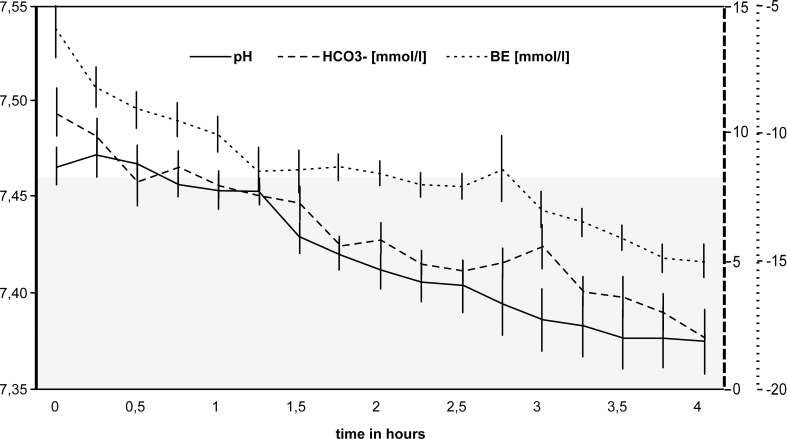
Changes in the acid-base balance during extracorporeal perfusion. Sodium bicarbonate buffer (HCO_3_^–^), base excess (BE) and pH-values all showed a steady decrease during the first 4 hours of extracorporeal perfusion. The pH stayed within the reference range without substitution (reference range 7.33–7.46, according to Baker *et al*. [19], grey area). The error bars indicate the standard error.

Changes in the acid-base balance of are illustrated in Fig 4. The pH stayed stable within the reference range for 4 hours (*P* = 0.063). The sodium bicarbonate buffer (HCO_3_^−^13.4 mmol/l, median) and base excess (BE –10.3, median) base values were reduced due to hemodilution and further decreased significantly during the 4-hour period (HCO_3_^−^5.1 mmol/l, BE –18.2, *P* = 0.031 after 4 hours).

Although steadily increasing from 4.34 to 5.61 mmol/l (*P* = 0.031), potassium levels were kept well within the reference range for Fischer rats (3.8–6 mmol/l [20]). As could be expected following hemodilution, hemoglobin base levels were significantly decreased (5.1 ± 2.3 mg/dl) in comparison with the reference values (14.9 ± 1.3 [20]). Since the composition of the perfusate was not changed, hemoglobin levels remained constant throughout the 4-hour perfusion period (*P* = 0.563).

### Assessment of Flap Perfusion

All 32 recipient rats survived the postoperative period and tolerated the anaesthesia and operative procedure well. Autocannibalism was observed to different degrees in all laboratory animals in group C2 (ischemia), but in no other study group. After commencing extracorporeal perfusion (ECP), a healthy pink color returned to the flap, and a capillary refill could be observed in all cases. These signs of flap viability could be maintained throughout the 8-hour perfusion interval. ICG angiography confirmed perfusion of the skin island flaps in groups C3 and ECP during the perfusion period. Weight gain of extracorporeally perfused flaps was 10% during the perfusion interval as opposed to 0% in the *in vivo* perfused flaps.

**Fig 5:**
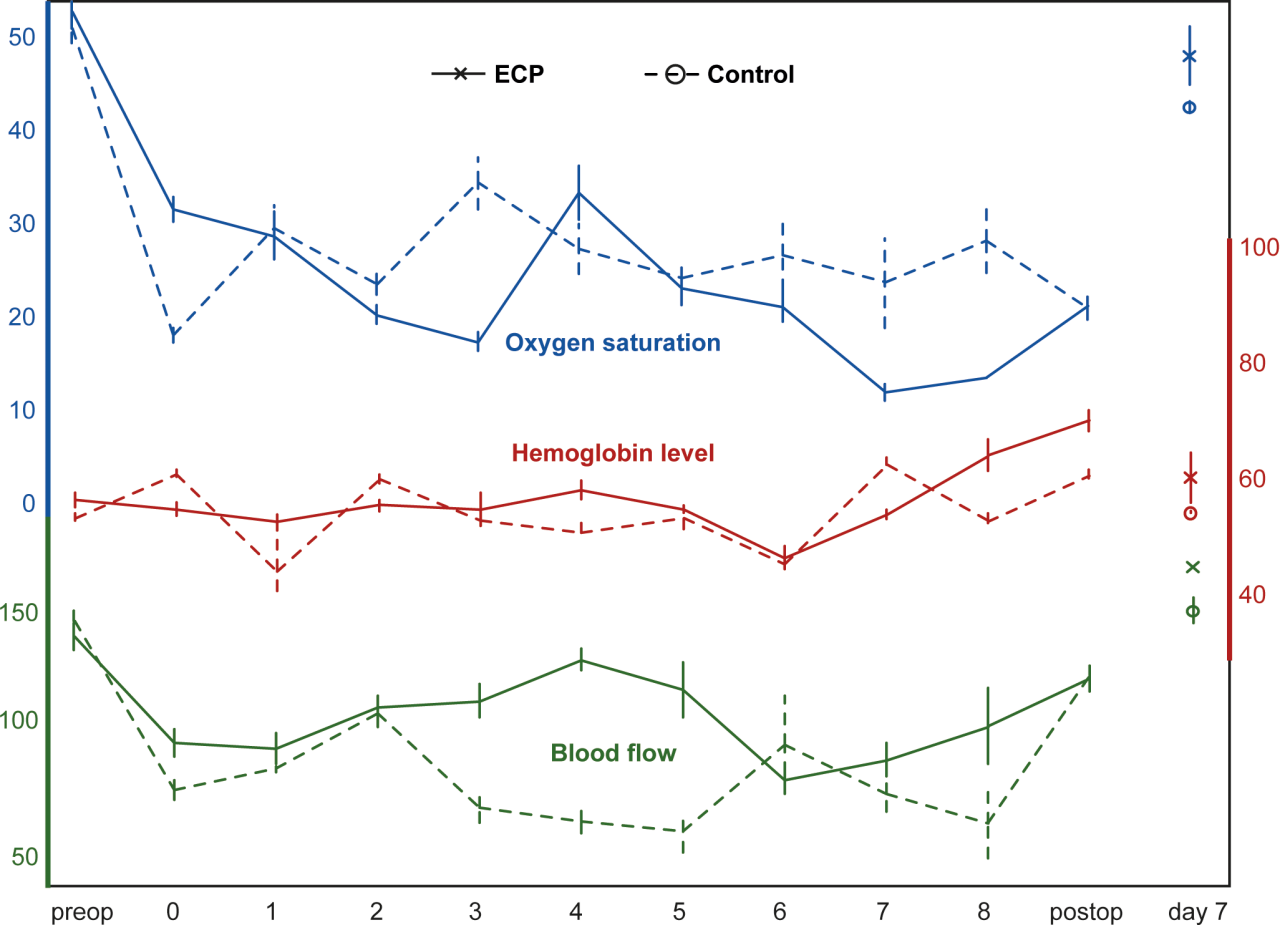
Combined laser Doppler flowmetry and remission spectroscopy. Depicted are the mean (superficial) laser spectrophotometric values for oxygen saturation (in percent), blood flow (in AU) and hemoglobin levels (in AU) over the experimental course. Measurements were conducted preoperatively (preop), after flap raise (0), during the course of the extracorporeal (group ECP) or *in vivo* (group C3) perfusion (1–8), postoperatively (postop) as well as at day 7. The error bars indicate the standard error.

O2C results are depicted in Fig 5 and Tables 3 and 4. The most comprehensive data were aquired in groups ECP and C3. Because of the analogous experimental setting, the comparison between these two groups also seems the most interesting to pursue. The curve progressions in both groups were remarkably similar. After flap raise and initiation of perfusion, a temporary decrease in oxygen saturation, blood flow and velocity was observed regardless of the perfusion method (extracorporeal or *in vivo*) in comparison with the initial values (before flap raise). In the further course of the perfusion, laser spectrophotometric values remained stable. Hemoglobin levels remained constant around 60 AU for 6 hours in both groups. Between hour 6 and 8, Hb raised slightly in both groups, but not statistically significant (ECP *P* = 0.375, K3 *P* = 0.999). At day 7, all laser spectrophotometric values were comparable with initial values in both groups. Due to high interindividual fluctuations of the method [21], no statistical comparisons between groups was performed. In the sham group (C1), as could be expected, all values were comparable to initial values at day 7, with the exception of an elevated hemoglobin level (*P* = 0.008). Intraoperative measurements were not performed in this group. While after 8-hour ischemia (group C2) only the oxygen saturation was significantly decreased (*P* = 0.008), all laser spectrophotometric values had significantly decreased by day 7.

**Table 3:** Laser spectrophotometric data.

**SO**_**2**_											
**Group**	**ECP**			**C1**		**C2**			**C3**		
**Time**	**0**	**8h**	**7d**	**0**	**7d**	**0**	**8h**	**7d**	**0**	**8h**	**7d**
**n**	8	8	6	8	8	8	8	8	8	8	5
**Med**	53	12	48	42	24	24	6	16	54	9	38
**Min**	35	5	24	18	2	11	2	0	22	5	34
**Max**	67	29	63	67	58	53	10	36	68	39	51
**Hb**											
**Group**	**ECP**			**C1**		**C2**			**C3**		
**Time**	**0**	**8h**	**7d**	**0**	**7d**	**0**	**8h**	**7d**	**0**	**8h**	**7d**
**n**	8	8	6	8	8	8	8	8	8	8	5
**Med**	56	66	58	53	61	71	79	64	55	60	55
**Min**	48	51	46	45	54	54	26	0	42	49	49
**Max**	75	87	83	65	70	78	94	91	70	74	64
**Flow**											
**Group**	**ECP**			**C1**		**C2**			**C3**		
**Time**	**0**	**8h**	**7d**	**0**	**7d**	**0**	**8h**	**7d**	**0**	**8h**	**7d**
**n**	8	8	6	8	8	8	8	8	8	8	5
**Med**	133	125	184	188	86	113	86	7	137	114	156
**Min**	57	33	128	116	44	56	53	0	77	34	133
**Max**	200	170	222	300	140	148	121	72	211	222	173
**Velocity**											
**Group**	**ECP**			**C1**		**C2**			**C3**		
**Time**	**0**	**8h**	**7d**	**0**	**7d**	**0**	**8h**	**7d**	**0**	**8h**	**7d**
**n**	8	8	6	8	8	8	8	8	8	8	5
**Med**	21	16	24	24	16	22	19	11	20	18	21
**Min**	12	10	19	20	11	17	14	0	18	13	18
**Max**	25	21	27	36	19	49	53	52	30	29	25

Descriptive statistics of (superficial) combined laser Doppler flowmetry and remission spectroscopy (O2C) values [oxygen saturation (SO_2_ in %), hemoglobin level (Hb in AU), blood flow (Flow in AU) and blood flow velocity (Velocity in AU)] of groups ECP (extracorporeal perfusion), C1 (sham operation), C2 (ischemia) and C3 (*in vivo* Perfusion) at the time points preoperatively (0), 8 hours (8h) and at day 7 (7d). The data are presented as total number of measured values (n), median (Med), minimum (Min) and maximum (Max) values.

**Table 4:** Comparative statistics of laser spectrophotometric data.

Group	ECP			C1	C2			C3		
Time	0–8h	0–7d	8–7d	0–7d	0–8h	0–7d	8–7d	0–8h	0–7d	8–7d
SO_2_	41 (0.125)	5 (1.000)	-36 (0.063)	19 (0.461)	18 (0.008*)	8 (0.102)	-10 (0.148)	45 (0.500)	16 (0.188)	-29 (0.063)
Hb	-10 (0.715)	-2 (0.345)	8 (0.219)	-8 (0.008*)	-8 (0.445)	7 (0.250)	15 (0.109)	-5 (1.000)	0 (0.500)	5 (0.625)
Flow	8 (0.125)	-51 (0.438)	-59 (0.156)	102 (0.945)	27 (0.078)	106 (0.008*)	79 (0.016*)	23 (0.500)	-19 (0.1000)	-42 (1.000)
Velocity	-4 (0.125)	-3 (0.563)	-8 (0.031*)	8 (0.945)	3 (0.469)	11 (0.016*)	8 (0.008*)	2 (0.500)	-1 (0.813)	-3 (0.813)

Comparative statistical assessment of (superficial) combined laser Doppler flowmetry and remission spectroscopy (O2C) values [oxygen saturation (SO_2_ in %), hemoglobin level (Hb in AU), blood flow (Flow in AU) and blood flow velocity (Velocity in AU)] of groups ECP (extracorporeal perfusion), C1 (sham operation), C2 (ischemia) and C3 (*in vivo* Perfusion). Wilcoxon’s test was used for statistical analysis between different time points [preoperatively (0), 8 hours (8h) and at day 7 (7d)]; *P* values < 0.05 were considered statistically significant (*).

### Assessment of Flap Viability at Day 7

**Fig 6:**
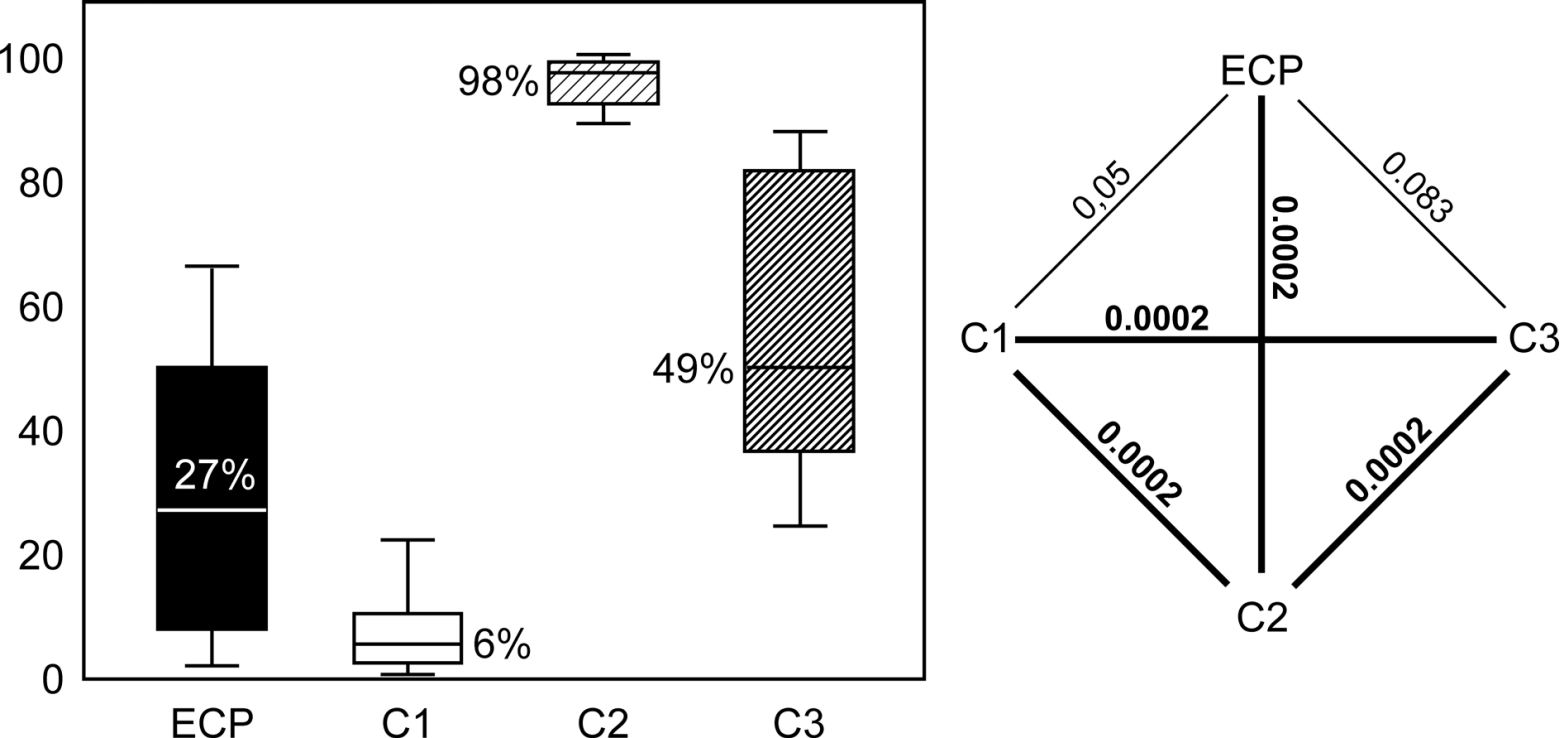
Rate of necrosis. Left: median percentage of necrotic area in groups ECP (8-hour extracorporeal perfusion), C1 (sham operation), C2 (8-hour ischemia) and C3 (8-hour *in vivo* perfusion). Right: graphical representation of statistical differences between the study groups. All significant (< 0.05) and highly significant (< 0.001) *P* values are printed bold. Kruskal-Wallis test followed by Mann Whitney U test were used for statistical analysis.

With 98%, 8-hour ischemia (C2) was associated with the highest rate of necrosis. Lowest rates were observed in in the sham group (C1), where only 6% of the transplanted tissue became necrotic. The rate of necrosis after 8-hour extracorporeal perfusion (27%) was significanly lower (*P* = 0.0002) than in group C2, while the difference between extracorporeal perfusion and both sham operation (*P* = 0.05) and *in vivo* perfusion (49%, *P* = 0.083) was not statistically different. All rates and *P* values are presented in Fig 6.

**Fig 7:**
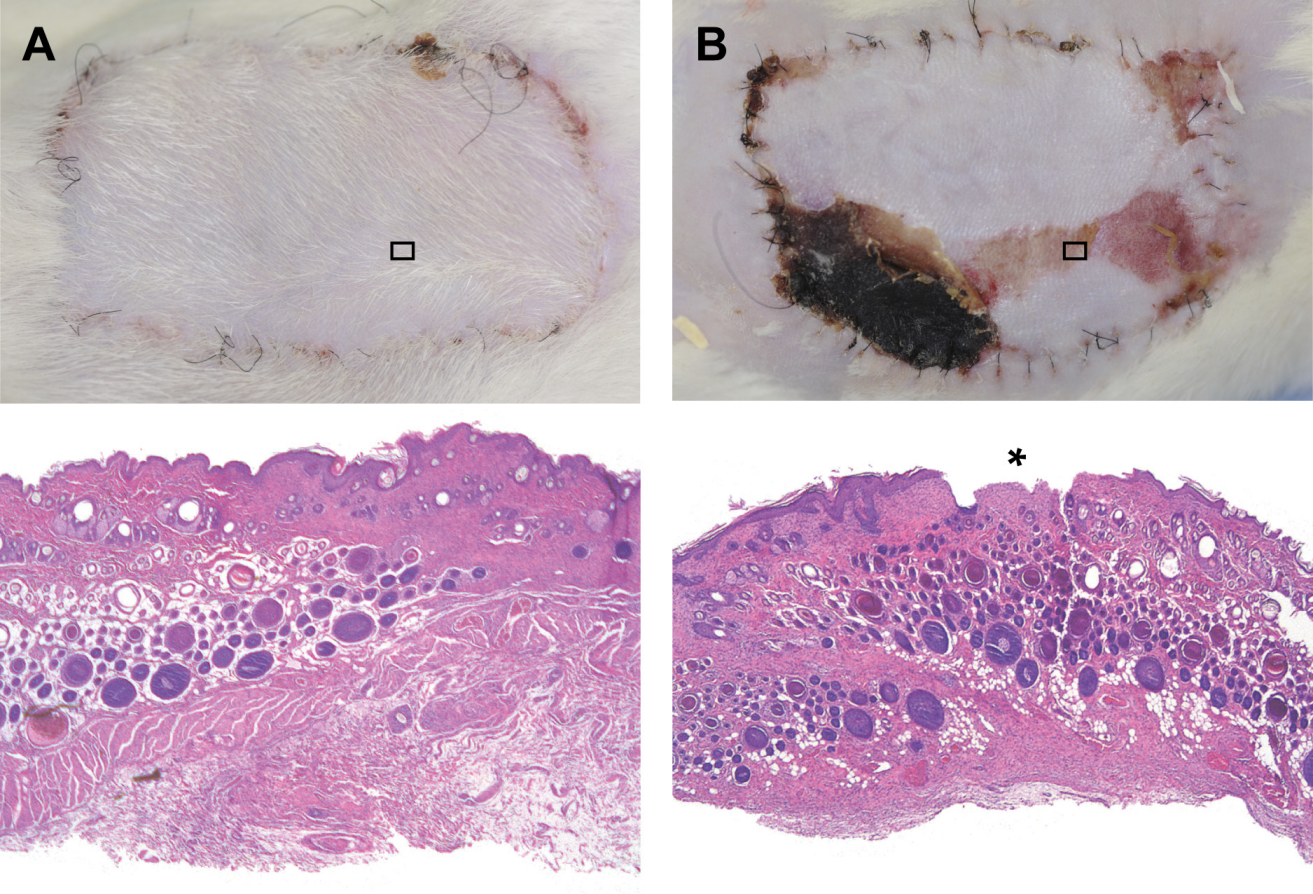
Clinical and histological images. (A) Completely healed in flap 7 days after 8-hour extracorporeal perfusion. Below: Histological image taken from the center of the same flap with normal tissue architecture and regular epithelium. (B) Partially necrotic epigastric flap 7 days after 8-hour extracorporeal perfusion. Below: Histologic image taken from an area with clinically apparent epitheliolyisis with histological disruption of the epithelium (*). HE staining, 30x magnification.

Structural analysis corroborated the clinical findings and the planimetric assessment of the rate of necrosis. Clinically healed flaps showed regular tissue architecture with preservation of endothelial integrity and without degeneration of skin, skin appendages, fatty tissue, blood vessels, muscle or lymph nodes, while necrotic areas showed typical signs of degeneration like disruption of the epithelium and fatty necrosis (Fig 7). Histologically, no signs of inflammation or bacterial contamination were observed.

## Discussion

The objective of this study was to develop an extracorporeal perfusion device for the prolonged vital storage of microvascular free flaps that should serve as a fundamental research tool for the investigation of extracorporeal free flap perfusion. The rat epigastric flap was chosen as animal model, a fascio-lipo-cutaneous flap, we have gained experience with in the past [22–24]. We report the successful transplantation of rat epigastric flaps after 8-hour extracorporeal perfusion. After 8-hour ECP and subsequent transfer, the majority of free flaps had healed in by day 7 with only minor partial necrosis (27%). Notably, ECP yielded even better results than *in vivo* perfusion (49%), although not statistically significant (*P* = 0,083). Blood flow through *in vivo* perfused flaps may have been temporarily compromised by low systemic blood pressure during deep anaesthesia and by vasospasms caused by manipulation of the pedicle vessels during flap raise or vessel preparation. The principle of extracorporeal perfusion, on the other hand, allows specifying exactly *when*, *what*, and *how much of it* enters a flap–without influencing the rest of the organism, and without being influenced by the rest of the organism. In our study, the use of an oxygenator and a controllable pump set at high physiological pressure and flow rates provided a constant flow of oxygenated blood to the raised flaps. Perfusate temperature and perfusate composition (heparinized, diluted blood) may also have helped to improve results in the ECP group: While maintaining the blood flow through the existing vascular network, oxygen consumption of the perfused flaps was reduced by allowing the perfusate to cool to room temperature (21 ± 1°C). In accordance with the law of Hagen-Poiseuille, dilution further improved the rheological properties of the perfusate. Finally, short-term *ex vivo* perfusion of free flaps with *heparinized* blood, as used in our study, is known to reduce the effect of ischemia-/ reperfusion damage [25]. Even though it remains unclear if these factors can be successfully transferred to long-term extracorporeal tissue perfusion, our results clearly indicate that short-term ECP can have a positive effect on flap viability.

Histological assessment confirmed the clinical observations, showing regular tissue architecture and intact vessel endothelium as well as intact epithelium in extracorporeally perfused flaps after one week. Considering the high success rate in the presented study, the cellular damage observed in other studies after prolonged perfusion [26–28] was either less substantial in our study or at least partially reversible.

Continuous evaluation of both perfusate and free flaps provided important insights on the pathophysiological processes during extracorporeal tissue perfusion. With initiation of the ECP, a clear capillary refill was observed. Venous return started about a minute later and the dark color of the blood impressively indicated the lower oxygen level of the venous return. As an indication for a maintained metabolism [29, 30], arterio-venous difference was 33.7% in our study (arterial 98.9%, venous 65.2%) and remained constant throughout the perfusion interval.

These results are substantiated by the O2C. After an initial decrease after flap raise, O2C values remained constant throughout the 8-hour ECP and reached base values 1 week after flap transplantation. The observed initial decrease, however, was not statistically significant. The temporal change of O2C values during extracorporeal perfusion was remarkably similar to *in vivo* perfused flaps (Fig 4), indicating that the probable causes for the initial decrease of O2C values were vasospasms or a changed vascular anatomy of the flaps after flap raise rather than an issues directly related to ECP. Except for group C2 (ischemia), all O2C values reached base values in all groups after revascularization had occurred by day 7. Dragu *et al*. [31] also observed a decrease of tissue oxygenation during extracorporeally perfused bovine rectus abdominis flaps, regardless of the perfusate used (crystalloid solution, whole blood). Since no active oxygenation was used in their study, however, oxygen levels were barely measurable (0–2%), underlining the importance of an active oxygenation [31].

Since no influence was taken on the composition of the perfusate, any changes in the composition are representative for the pathophysiological and biochemical changes inside the flap. Analogous to Constantinescu *et al*. [8], constantly high oxygen saturations and oxygen partial pressures were measured during ECP despite significant hemodilution and the use of ambient air (21% oxygen) instead of pure oxygen. Apart from vasospasms and altered anatomy after flap raise, the discrepancy between constantly high oxygen values in the *perfusate* and a decreased oxygen saturation in the perfused *flaps* may be explained by the physical relationship between temperature and hemoglobin oxygen binding capacity: Hypothermia increases the affinity of hemoglobin to bind oxygen (hence the high oxygen levels in the perfusate) but also leads to a decreased tendency to release oxygen to the tissue, which may explain the decreased tissue oxygenation. Hypothermia decreases oxygen consumption and energy turnover in living tissues and is therefore commonly used for the vital storage of organs and tissues [8, 32]. Like most authors who used whole blood for tissue perfusion [25, 28, 31, 33–35], we allowed the perfusate temperature to drop to room temperature. The high success rate in our study suggests that the reduced oxygen delivery could at least partially be compensated by the simultaneous decrease of metabolic activity in the tissue. However, further scientific effort is needed to illuminate the effect of temperature during extracorporeal tissue perfusion.

The high quality of the perfusion in our study was further confirmed by high, constant pH level and potassium levels that stayed within normal range throughout the measurement period. (Metabolic) Acidosis [36–38] and high potassium levels [8, 38] are common signs of hypoxia observed during ECP. In our study, no supplementation of sodium bicarbonate [36–38] was necessary to avoid acidosis and neither insulin nor glucose had to be administered to decrease potassium levels. Despite normal pH and potassium levels in our study, corbon dioxide partial pressures, bicarbonate and base excess, however, decreased significantly during perfusion. This acid-base inbalance, referred to as *respiratorically compensated metabolic acidosis*, is usually caused by lactate acidodis in the course of ischemia- and hypoxia-related reduced perfusion. As the results from the preliminary study showed, lactate (and LDH) levels both increased significantly due to progressive hemolysis. It seems safe to assume that intravasal hemolysis has played a decicive role in the development of the observed acid-base dysbalance.

During extracorporeal tissue perfusion, basic bodily functions like respiration, removal of metabolites, acid-base balance and temperature regulation have to be maintained by a decoupled circulation system. Apart from the “mechanical organs” (oxygenator, pump), the *perfusate* plays a central role in maintaining these conditions. Oxygenated whole blood, which remains the gold standard in cardiac surgery [39] and and organ transplantation [11], was the obvious choice in our study since it provides an oxygen transport system (hemoglobin), elaborate buffer systems (bicarbonate, phosphate, proteinate) and even limited immunological activity. The main drawbacks of blood as perfusate are hemolysis and hemostasis. Both phenomena can lead to occlusion of tubes or vessels, compromising the perfusion and eventually leading to partial or total flap loss. Contact of blood with artificial surfaces (i.e. silicone tubes) leads to hemostatis requiring heparinization. This in turn poses the risk of uncontrolled bleeding from the flap. Finding a balance between these two risks remains an important challenge when using whole blood as perfusate. The cellular blood components can be damaged by roller pumps, pulsatile pumps and membrane pumps and lead to hemolysis within hours [40]. Despite the fact that blood was used as perfusate in most tissue perfusion studies [8, 11, 28, 31, 32, 34, 36], the issue of hemolysis was only broached by two research groups [11, 29]. Analogous to our own experiments, Mayer *et al*. [29] observed a photometric haemoglobin increase within 4.5 hours as an indicator for progressive hemolysis. The consequence we drew from the literature and our preliminary study was to replace the perfusate every 4 hours to avoid a negative effect caused by progressive hemolysis. For an 8-hour perfusion this meant a single perfusate change, which was acceptable. Due to the limited availability of blood, alternatives need to be considered for longer perfusions in small animal models. Worner *et al*. [11] recently described a pulsatile pump system for the perfusion of small animal organs and composite tissues of which the authors claim it would lead to less mechanical damages to erythrocytes in comparison with conventional roller pumps. However, no data are presented with regard to hemolysis and it remains unclear on what scientific basis these assumptions were made. Although kidney perfusion was clearly in the focus of their study, the authors achieved to perfuse 5 out of 7 rabbit epigastric fat flaps over a period of 5 days. In 2 cases, excessive bleeding occurred in the first 24 hours and perfusion was terminated. In contrast to our study, however, Worner et al. did not attempt to re- or transplant the perfused tissue, so the clinical success of the perfusion can ultimately not be confirmed.

### Perspectives

Despite numerous issues that still need to be addressed in further studies, the phenomenon of a decoupled circulation does offer some compelling advantages, like improved monitoring capabilities, flap conditioning by intermittend perfusion and the possibility to manipulate various pyhsiological aspects by changing pressure, flow or perfusate composition. Moreover, local administration of drugs, hormons or gene transfer becomes possible, without influencing the body’s circulation. This opens a wide field of new exciting clinical applications including prolonged vital storage (as addressed in this study), bridging time to transplant [11], saving laboratory animals [41], local chemotherapy in sarcomas [42] or melanomas [43], tissue engineering [44, 45] or simulation of realistic preparation conditions for the training of reconstructive surgeons [12]. Born out of necessity, we would like to address yet another theoretical clinical application of extracorporeal tissue perfusion. Founded on the knowledge that transplanted composite tissues eventually become independent from the supplying vessels [24, 34, 46], temporary ECP of transplanted free flaps may hold the key to free composite tissue transplantation without anastomosis. This would potentially allow complex reconstructions even in otherwise desolate cases, like in the irradiated, vessel depleted neck.

## Conclusions

This study constitutes an important input to the understanding of the processes in living composite tissues during extracorporeal perfusion and offers a validated experimental model for further research. With this experimental setup, successful replantation after 8-hour extracorporeal perfusion of rat epigastric flaps was possible. Notably, results after temporary extracorporeal perfusion were superior to *in vivo* perfusion. This observation may be explained by maintaining a constant flow of oxygenated, heparinized blood to the raised flaps while provoking a hypothermia-induced downregulation of the flaps’ oxygen consumption. Extracorporeal tissue perfusion remains a highly complex procedure, influenced by a myriad of paramters like the different ischemia tolerance of different tissues, the choice of perfusate, optimal oxygen/ carbondioxide ratio, perfusion pressures and temperature. The investigation of these fundamental questions, however, is exacly what the described perfusion system and animal model was developed for.

## Supporting Information

S1 Fig Planimetric measurement of rate of necrosis. *Upper row*: Step-by-step assessment of the rate of necoris. ImageJ was used to assess the total flap area (in pixels), as well as the area of all viable parts of the flap and all necrotic parts. Dividing the pixel values (necrotic area/ total flap area*100%) results in the percentage area of necrosis (= rate of necrosis). *Lower row*: representative images of all experimental groups (ECP = 8-hour extracorporeal perfusion), sham operation (C1), 8-hour ischemia (C2), 8-hour *in vivo* perfusion (C3)).
(TIF)

S2 Fig Indocyanine green angiography during extracorporeal perfusion. (A) Rat in supine position with femoral catheter (fc) in place. The asterisk (*) marks the reference area with maximal indocyanine green (ICG) intensity. (B) ICG was administered directly into the venous reservoir, circulated through the tubing system, passed the membrane oxygenator (om) and accumulated in the arterial reservoir (ar). (C) Extracorporeally perfused epigastric flap (ef) with high ICG-signal in artery (a), vein (v) and throughout the whole skin island of the flap, indicating maintained flap perfusion. ICG is metabolized in the liver (li), which leads to a high ICG signal in this area.
(TIF)
