# Supplementary figures and images for: Development of an Extracorporeal Perfusion Device for Small Animal Free Flaps
Source: PLoS One. 2016 Jan 25;11(1):e0147755. doi: 10.1371/journal.pone.0147755 (PMC4726627; doi:10.1371/journal.pone.0147755)

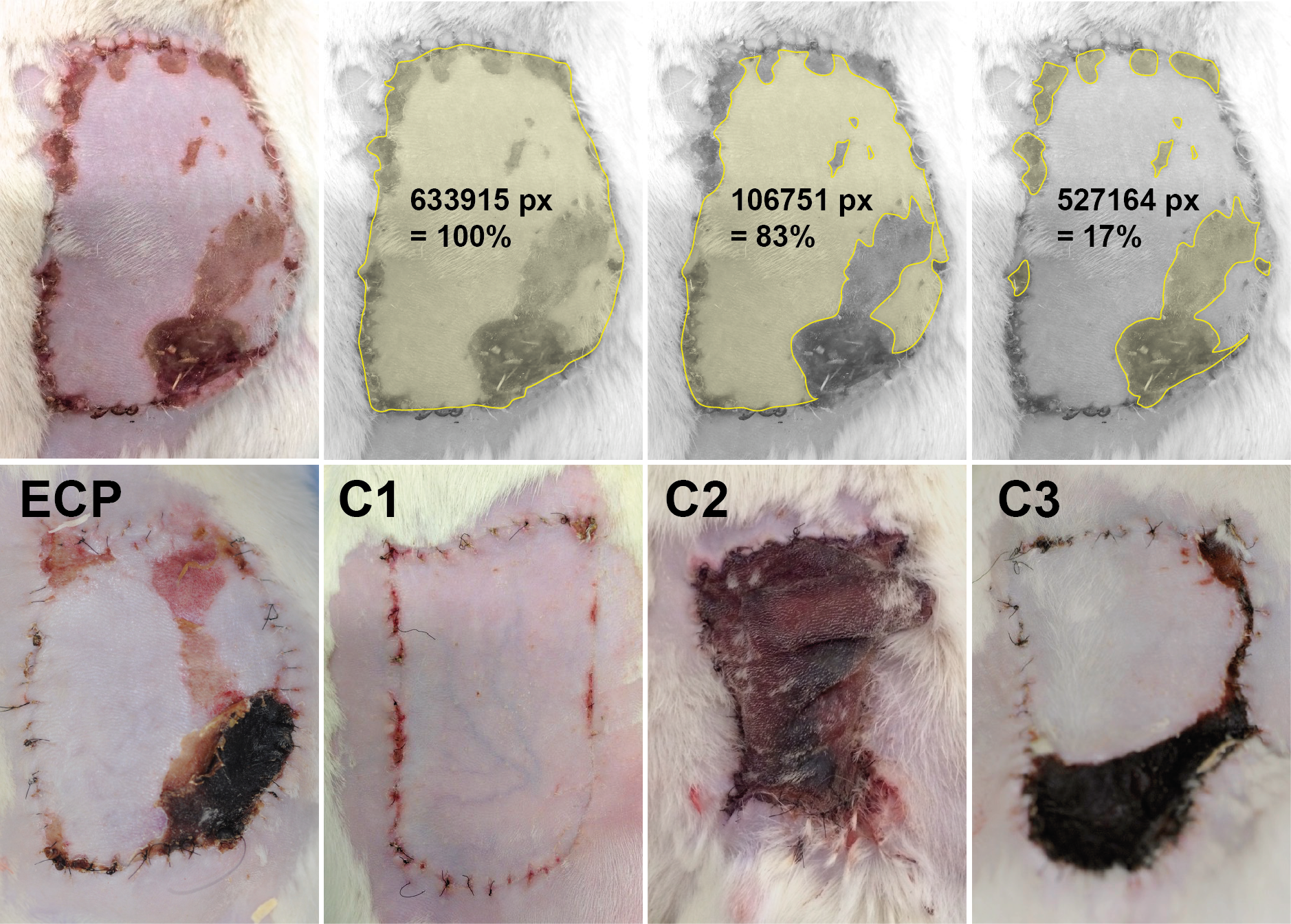

Supplement: S1 Fig — Upper row: Step-by-step assessment of the rate of necoris. ImageJ was used to assess the total flap area (in pixels), as well as the area of all viable parts of the flap and all necrotic parts. Dividing the pixel values (necrotic area/ total flap area*100%) results in the percentage area of necrosis (= rate of necrosis). Lower row: representative images of all experimental groups (ECP = 8-hour extracorporeal perfusion), sham operation (C1), 8-hour ischemia (C2), 8-hour in vivo perfusion (C3)). (TIF) [file pone.0147755.s001.tif]

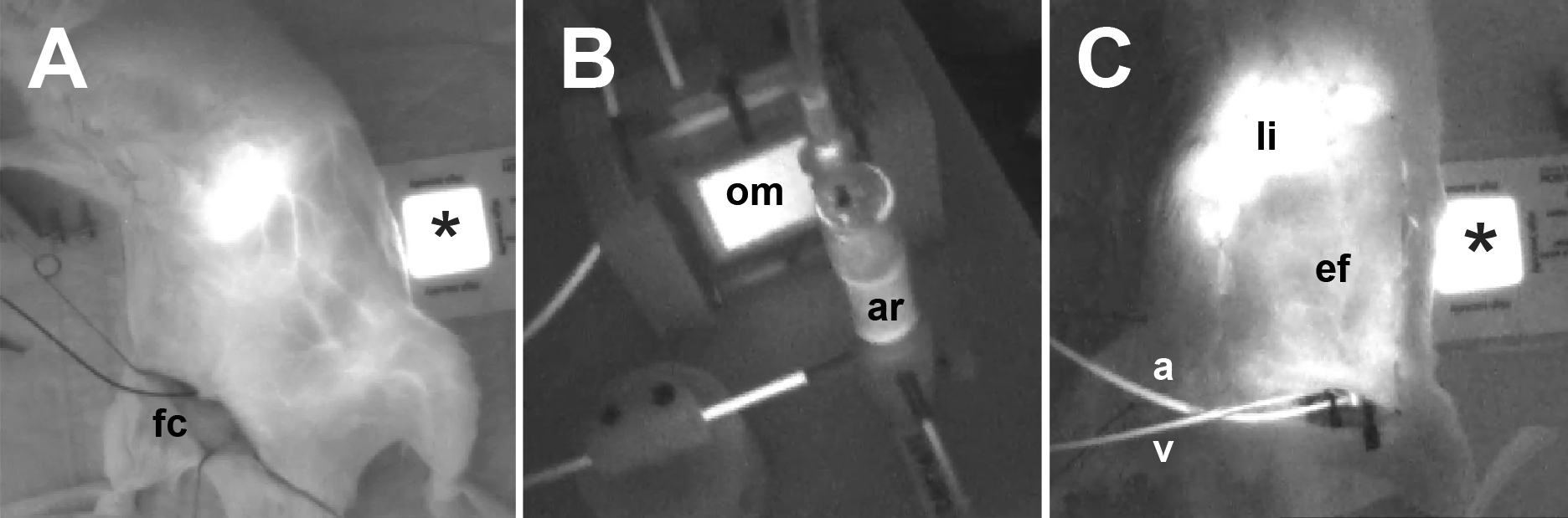

Supplement: S2 Fig — (A) Rat in supine position with femoral catheter (fc) in place. The asterisk (*) marks the reference area with maximal indocyanine green (ICG) intensity. (B) ICG was administered directly into the venous reservoir, circulated through the tubing system, passed the membrane oxygenator (om) and accumulated in the arterial reservoir (ar). (C) Extracorporeally perfused epigastric flap (ef) with high ICG-signal in artery (a), vein (v) and throughout the whole skin island of the flap, indicating maintained flap perfusion. ICG is metabolized in the liver (li), which leads to a high ICG signal in this area. (TIF) [file pone.0147755.s002.tif]
